# Knockdown of the TP53-Induced Glycolysis and Apoptosis Regulator (TIGAR) Sensitizes Glioma Cells to Hypoxia, Irradiation and Temozolomide

**DOI:** 10.3390/ijms20051061

**Published:** 2019-03-01

**Authors:** Gabriele D. Maurer, Sonja Heller, Christina Wanka, Johannes Rieger, Joachim P. Steinbach

**Affiliations:** 1 Dr. Senckenberg Institute of Neurooncology and University Cancer Center (UCT), University Hospital Frankfurt, Goethe University, 60590 Frankfurt am Main, Germany; gabriele.maurer@kgu.de (G.D.M.); sonja.heller@kgu.de (S.H.); christina.wanka@gmx.de (C.W.); j.rieger@uni-tuebingen.de (J.R.); 2 German Cancer Research Center (DKFZ) Heidelberg, and German Cancer Consortium (DKTK), Partner Site Frankfurt/Mainz, 60590 Frankfurt am Main, Germany; 3 Interdisciplinary Division of Neuro-Oncology, Hertie Institute for Clinical Brain Research, University Hospital Tuebingen, Eberhard Karls University, 72076 Tuebingen, Germany

**Keywords:** TP53-induced glycolysis and apoptosis regulator, glioma, hypoxia, irradiation, temozolomide, reactive oxygen species, hypoxia-inducible factor

## Abstract

The TP53-induced glycolysis and apoptosis regulator (TIGAR) has been shown to decrease glycolysis, to activate the pentose phosphate pathway, and to provide protection against oxidative damage. Hypoxic regions are considered characteristic of glioblastoma and linked with resistance to current treatment strategies. Here, we established that LNT-229 glioma cell lines stably expressed shRNA constructs targeting *TIGAR*, and exposed them to hypoxia, irradiation and temozolomide. The disruption of *TIGAR* enhanced levels of reactive oxygen species and cell death under hypoxic conditions, as well as the effectiveness of irradiation and temozolomide. In addition, *TIGAR* was upregulated by HIF-1α. As a component of a complex network, TIGAR contributes to the metabolic adjustments that arise from either spontaneous or therapy-induced changes in tumor microenvironment.

## 1. Introduction

Glioblastoma is a highly aggressive and lethal primary brain tumor. The current standard therapy includes maximal safe surgical resection and radiotherapy, plus concomitant and adjuvant temozolomide, an oral alkylating agent. Despite these efforts, tumor recurrence commonly occurs within 12 months after primary treatment. Understanding the mechanisms underlying glioma behavior and resistance to therapy is essential for the development of new therapeutic strategies.

Integrated genomic analysis has allowed us to distinguish glioblastomas into the proneural, neural, classical, and mesenchymal subtypes [1]. Further analysis has revealed a discordance between genomic features and proteomic activation status, and led to the notion of a complex interplay between signaling and molecular alterations [2]. Especially the mesenchymal phenotype, representing approximately 20% of glioblastomas, has been associated with more aggressive disease as opposed to the proneural phenotype. The acquisition of mesenchymal attributes is thought to confer treatment resistance and invasiveness [3,4]. Mesenchymal glioblastoma cells often harbor mutations and/or deletions in the gene encoding neurofibromin 1 (*NF1*). The cell line LNT-229, used in the following experiments, has been reported not to express *NF1* due to gene deletion [5].

The TP53-induced glycolysis and apoptosis regulator (TIGAR) exhibits phosphatase activity on, among others, fructose-2,6-bisphosphate, fructose-1,6-bisphosphate [6], and 2,3-bisphosphoglycerate [7]. It has been shown to decrease glycolysis and levels of reactive oxygen species (ROS) [8], contributing to the metabolic adjustments mediated by TP53. In tumors originating from different tissues, an overexpression of *TIGAR* has been described. In chronic lymphocytic leukemia [9], in cytogenetically normal acute myeloid leukemia [10], and in lung adenocarcinoma [11], high *TIGAR* expression and TIGAR protein, were associated with shorter survival.

In the glioma cell lines U87MG and T98G, an increase in TIGAR was observed following irradiation and suppression of irradiation-induced TIGAR by RNA interference reduced colony-forming capacities [12]. Cell density, as assessed by crystal violet staining applied over a period of five days, was reduced in cells treated with siRNA targeting *TIGAR* [12]. In the glioma cell lines A172 and T98G, radiosensitization induced by siRNA-mediated *TIGAR* gene silencing correlated with an inhibition of thioredoxin-1 (TRX1) nuclear translocation [13]. In an orthotopic mouse model using U87MG glioma cells, *TIGAR* knockdown prolonged the survival of tumor-bearing mice and enhanced the radiosensitivity of xenografts overexpressing thioredoxin reductase-1 (TRXR1) [14]. Furthermore, stable *TIGAR* knockdown slightly decelerated HepG2 xenograft growth and enhanced the inhibitory effect of epirubicin treatment on tumor size [15]. A549 non-small cell lung cancer cells were more sensitive to etoposide, 5-fluorouracil, and cis-dichlorodiamineplatinum when transfected with siRNA-targeting *TIGAR* [16].

Using glioma cell lines LNT-229 and T98G, we previously observed that the ectopic expression of *TIGAR* attenuated cell death induced by glucose and oxygen restriction, lowered ROS levels, and raised concentrations of reduced glutathione [17]. TIGAR might therefore facilitate resistance to radio- and chemotherapy, both of which induce the formation of ROS [18,19]. In order to assess the impact of *TIGAR* depletion on the efficacy of current therapeutic strategies, we generated LNT-229 cells that stably expressed shRNA against *TIGAR* and subjected them to irradiation and temozolomide.

## 2. Results

### 2.1. HIF-1α Upregulates TIGAR Expression in Both Normoxia and Hypoxia

**Figure 1:**
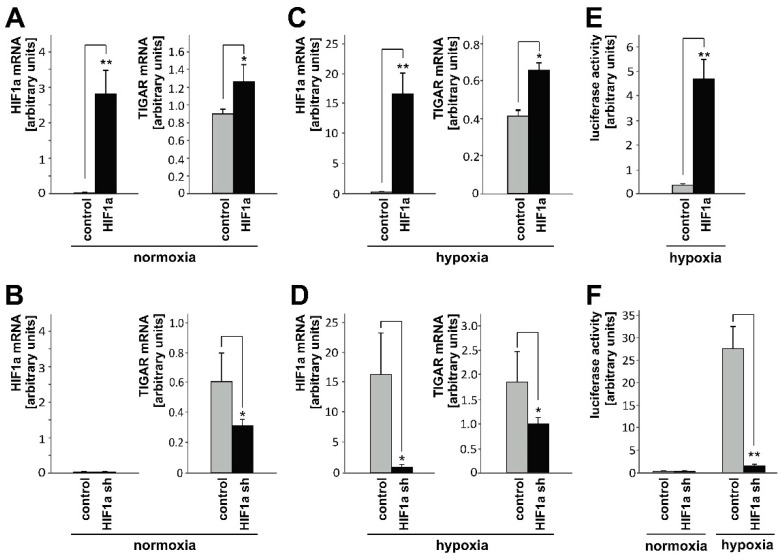
LNT-229 cells were transiently transfected with pcDNA3-HIF-1α or pcDNA3 control and subjected to normoxia (**A**) and hypoxia (**C**) 24 h later. Another 24 h later, *HIF-1α* and the TP53-induced glycolysis and apoptosis regulator (*TIGAR*) expression was analyzed by RT-qPCR (mean + SD, * *p* < 0.05, ** *p* < 0.01). LNT-229 cells that stably expressed shRNA targeting *HIF-1α* or its Drosophila homolog *Sima* (control), were grown in normoxia (**B**) and hypoxia (**D**) and 24 h later checked for *HIF-1α* and *TIGAR* mRNA content (mean + SD, * *p* < 0.05) (**E**, **F**). To verify the successful modulation of HIF-1α, HIF-specific transcriptional activity was examined by luciferase reporter assay (mean + SD, ** *p* < 0.01).

Hypoxic regions are considered characteristic of glioblastoma, are linked to a stabilization of hypoxia-inducible factors (HIFs) and are probably related to resistance to current treatment strategies, i.e. radio- and chemotherapy [20,21]. The LNT-229 cells transiently transfected with a plasmid encoding *HIF-1α*, displayed higher *TIGAR* levels than the corresponding control cells (Figure 1A,C), whereas knockdown of *HIF-1α* was associated with lower expression of *TIGAR* (Figure 1B,D). The functionality of *HIF-1α* modulation was examined by a luciferase reporter assay (see the 3HRE-pTK-luc construct, Figure 1E,F). Hypoxia increased HIF-specific transcriptional activity, and *TIGAR* gene suppression attenuated this rise. In the tumor microenvironment, such a positive feedback loop might help to restrain ROS levels under hypoxic conditions.

### 2.2. TIGAR Knockdown Involves Higher ROS Levels in Hypoxia

**Figure 2:**
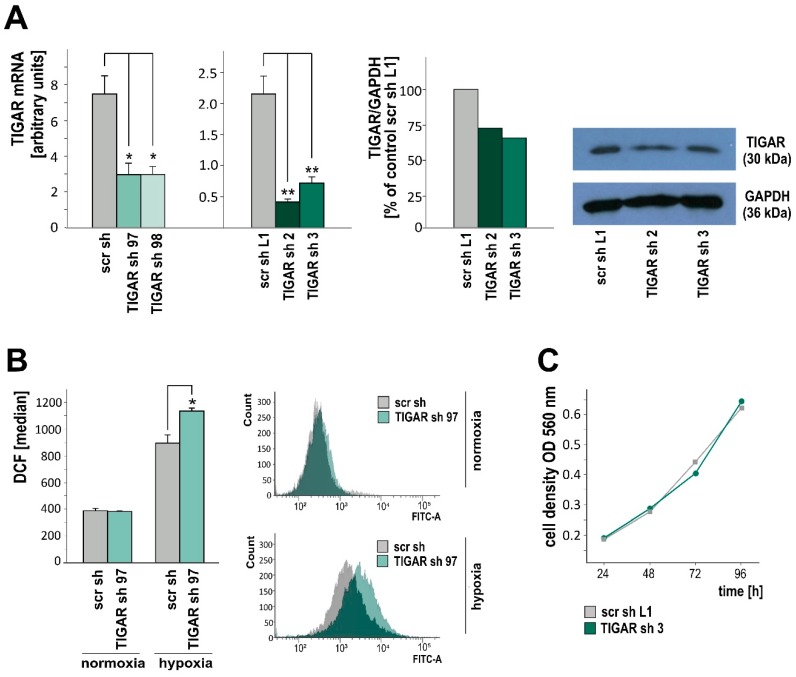
(**A**) shRNA-mediated *TIGAR* attenuation was verified by RT-qPCR (left, mean + SD, * *p* < 0.05, ** *p* < 0.01) and western blot analysis (center, densitometric analysis, right, film). (**B**) LNT-229 cells stably expressing a scrambled shRNA sequence (scr sh) or shRNA targeting *TIGAR* (TIGAR sh 97) were cultured in serum-free medium supplemented with 5 mM glucose under normoxic or hypoxic conditions for 24 h. Intracellular ROS were determined using H_2_DCFDA and flow cytometry (median fluorescence intensity, mean + SD, * *p* < 0.05). (**C**) LNT-229-shTIGAR (TIGAR sh 3) and control (scr L1) cells were grown in normoxia. After 24 h, 48 h, 72 h and 96 h, crystal violet staining was performed to evaluate cell density.

Following our experiments with overexpression and transient knockdown [17], we established LNT-229 glioma cells that stably expressed shRNA against *TIGAR*. Knockdown was verified by RT-qPCR and western blot analysis (Figure 2A). The ROS, which are byproducts of aerobic metabolism, regulate diverse signaling pathways and hereby enable cells to adapt to environmental changes in order to grow and to survive [22]. However, ROS may induce DNA damage causing genomic instability and, in higher amounts, initiate cell death [23]. Therefore, the reduction–oxidation (redox) function is an essential component of the cell’s homeostatic network. In line with previous reports [8,17], a lower expression of *TIGAR* was associated with higher ROS levels under hypoxic conditions. In normoxia, the depletion of *TIGAR* did not alter intracellular ROS concentrations, as assessed by the ROS-sensitive dye dichlorodihydrofluorescein diacetate (H_2_DCFDA), and flow cytometry (Figure 2B). Likewise, cell density was not affected by *TIGAR* knockdown (Figure 2C).

### 2.3. TIGAR Gene Silencing Enhances Cell Death Associated with Oxygen Restriction

**Figure 3:**
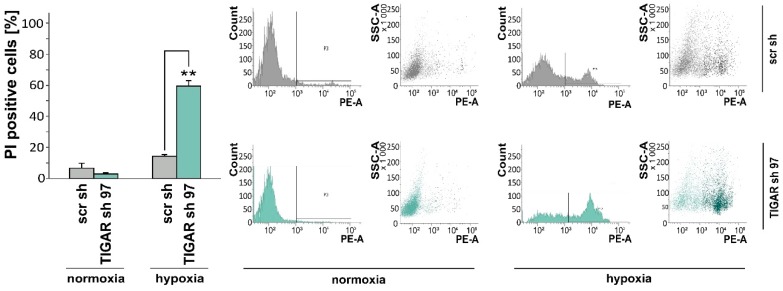
LNT-229-shTIGAR (TIGAR sh 97) and control (scr sh) cells were cultured in serum-free medium supplemented with 2 mM glucose for 36 h. Thereafter, cell death was assessed by propidium iodide staining and flow cytometry (mean percentage of PI-positive cells + SD, ** *p* < 0.01).

ROS are generated in several subcellular compartments and increase under hypoxia. The precise mechanism by which hypoxia enhances ROS remains to be elucidated, but presumably the mitochondria electron transport chain plays a key role [24,25]. The inhibition of intracellular ROS production and/or intensification of ROS clearance are considered necessary to promote cell survival during and after oxidative stress [26]. Rapidly expanding tumors, e.g., glioblastomas, are characterized by a fluctuating availability of oxygen and nutrients such as glucose, and malignant progression is supposed to arise from the adaptation to these conditions [27]. In order to mimic that situation, we subjected the cells to serum, glucose, and oxygen restriction. Using propidium iodide (PI) staining and flow cytometry, we observed that *TIGAR* knockdown promoted cell death under hypoxic conditions. In normoxia, fractions of PI-positive cells did not differ significantly between LNT-229-shTIGAR and control cells (Figure 3).

### 2.4. TIGAR Knockdown Increases Vulnerability to Ionizing Radiation

**Figure 4:**
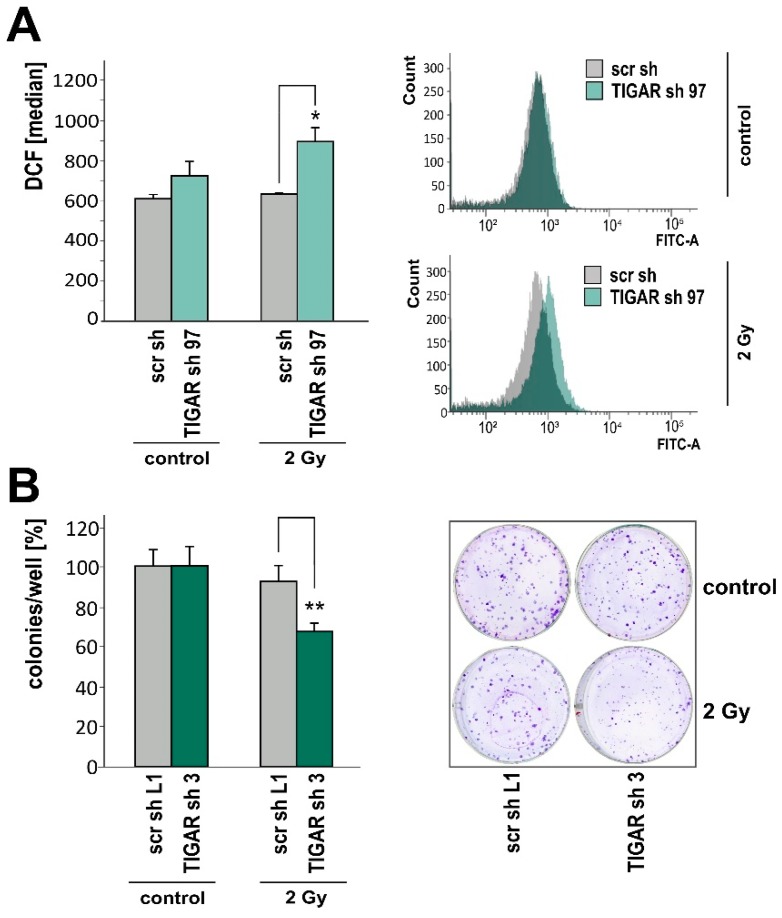
(**A**) LNT-229-shTIGAR (TIGAR sh 97) and control (scr sh) cells were cultured in medium supplemented with 10% FCS and 25 mM glucose for 24 h and then irradiated with a photon dose of 2 Gy. After 6 h, ROS levels were determined (median fluorescence intensity, mean + SD, * *p* < 0.05). (**B**) LNT-229-shTIGAR (TIGAR sh 3) and control (scr sh L1) cells were seeded at low density (500 cells per six-well), 24 h later irradiated, and then monitored for clonogenic survival (left, mean number of the colonies, displayed as percentages of the controls, + SD, ** *p* < 0.01) (right, representative photo of the colonies stained with crystal violet in six-wells).

Ionizing radiation, which can be involved in both the induction and treatment of tumors, prompts the generation of ROS [28]. Due to an increased availability of free radical scavengers or metabolic quiescence, low ROS concentrations help to protect cell genomes from endogenous and exogenous oxidative stress-mediated damage [29]. Using H_2_DCFDA and flow cytometry, we observed an increase in ROS following *TIGAR* gene silencing and exposure to 2 Gy irradiation (Figure 4A). In addition, clonogenic survival subsequent to irradiation was reduced in LNT-229-shTIGAR cells when compared to control cells. Non-irradiated LNT-229-shTIGAR and control cells exhibited similar levels of clonogenic survival (Figure 4B).

### 2.5. TIGAR Depletion Boosts the Effects of Temozolomide on Cell Density and Clonogenicity

**Figure 5:**
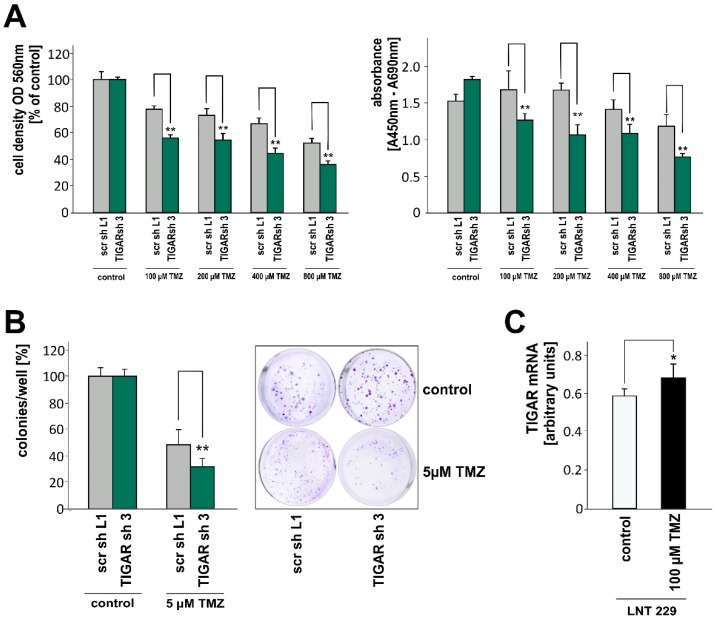
(**A**) LNT-229-shTIGAR (TIGAR sh 3) and control (scr sh L1) cells were exposed to temozolomide (TMZ) in serum-free medium. After 24 h, adherent cells were stained with crystal violet (left, mean absorbance, displayed as percentages of controls, + SD, ** *p* < 0.01). In parallel, BrdU incorporation was determined (right, mean + SD, ** *p* < 0.01). (**B**) Cells were treated with temozolomide, and assessed for clonogenic survival (mean number of colonies, displayed as percentages of controls, + SD, ** *p* < 0.01). (**C**) Following treatment with temozolomide, *TIGAR* expression was analyzed in LNT-229 cells (mean + SD, * *p* < 0.05).

Since at least 2005, temozolomide has been an integral component of glioblastoma treatment [30], and the induction of apoptosis by temozolomide has been linked to a ROS burst [31,32]. LNT-229-shTIGAR cells, presumably exhibiting less ROS clearance, were found to be more sensitive to temozolomide exposure than control cells, as reflected by the lower cell density and by the lower BrdU incorporation (Figure 5A). As acute cell behavior and clonogenicity require distinct conditions [33], we also looked at colony formation and found that the reproductive ability of the cells was impaired by *TIGAR* knockdown, too (Figure 5B). Underlining the potential biological relevance of TIGAR, treatment with temozolomide increased *TIGAR* expression in parental LNT-229 cells (Figure 5C).

### 2.6. Attenuation of TIGAR is Accompanied by Metabolic Changes

**Figure 6:**
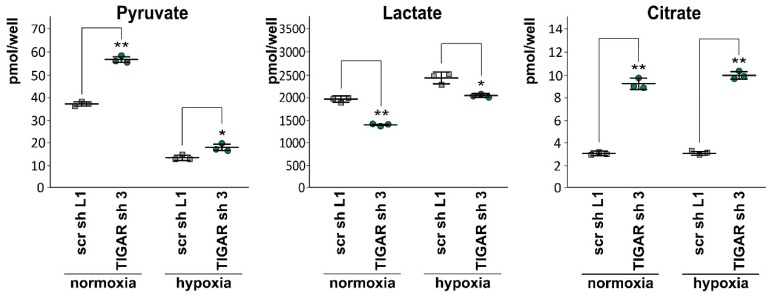
LNT-229-shTIGAR (TIGAR sh 3) and control (scr sh L1) cells were cultured in serum-free medium supplemented with 2 mM glucose for 8 h. Subsequently, the contents of pyruvate, lactate, and citrate in the supernatants were measured (mean + SD, * *p* < 0.05, ** *p* < 0.01).

Under certain circumstances, *TIGAR* overexpression can promote cellular respiration [17,34]. Recently, when exploring the role of TIGAR in skeletal muscle, a decrease in mitochondrial number and oxidative phosphorylation was described in *TIGAR* knockout mice compared with wild-type mice [35]. However, extensive metabolic analysis was not the purpose of the present study. In order to gain an impression of the alterations induced by *TIGAR* knockdown, we determined the extracellular concentrations of pyruvate, lactate, and citrate. Both in normoxia and in hypoxia, the levels of pyruvate and citrate in supernatants were found to be higher than in those of the LNT-229 cells expressing scrambled shRNA. By contrast, extracellular lactate was reduced following *TIGAR* gene suppression (Figure 6).

## 3. Discussion

TIGAR has been described as directing flux through the oxidative pentose phosphate pathway and as supporting the cell’s antioxidant defense (for a comprehensive review, please refer to [36]). In mouse intestinal adenoma models, *TIGAR* not only promoted regeneration of healthy tissue, but also supported tumor development [37]. Further, in nasopharyngeal carcinoma [38] and non-small cell lung cancer [39], TIGAR has been linked with enhanced invasion and metastasis. The antiproliferative, antimigratory, and proapoptotic effects of dioscin on hepatocellular carcinoma have been related to an inhibition of TIGAR [40]. In any case, in a pancreatic ductal adenocarcinoma model, loss of *TIGAR* did not improve survival, and even amplified metastatic spread [41], and in colorectal cancer patients, a higher TIGAR plasma level was associated with lower risk of metastasis [42]. Using LNT-229 glioma cells, we demonstrated that stable *TIGAR* gene silencing (1) was associated with higher ROS levels under hypoxic conditions, (2) increased cell death in hypoxia, and (3) sensitized cells to radiation and temozolomide. Besides, we showed that *TIGAR* was positively regulated by HIF-1α.

In 2006, *TIGAR* was identified as a TP53 target gene [8]. However, in several tumor entities, high TIGAR expression has been observed independent of TP53 function [43]. Depending on the cell type and environmental conditions, HIF-1 may also induce *TIGAR* expression. The p300/CBP-associated factor (PCAF), a common transcriptional coactivator, presumably controls the differential recruitment of TP53 and HIF-1α to the promoters of *TIGAR* and other metabolic regulators [44]. Alleviating oxidative stress and glycolysis, TIGAR has distinct effects on the cell cycle, senescence, and survival, which are partially based on cell type, cellular metabolism, and the extent of injury. Hypoxia, irradiation, and temozolomide can upregulate TP53 [45,46,47]. Hypoxia and irradiation are known to stabilize HIF-1α [48,49,50]. TP53 and HIF-1α interact with each other both directly and indirectly [51]. Both TP53 and HIF-1α may ultimately upregulate TIGAR, which in turn contributes to restraining ROS levels.

The enhanced susceptibility of LNT-229-sh TIGAR cells to radiation and temozolomide is in line with earlier reports. Peña-Rico et al. described a radiosensitization of glioma cells upon *TIGAR* knockdown [12]. In tumor cell lines of non-glial origin, *TIGAR* suppression enhanced the effect of the antineoplastic agents epirubicin, 5-fluorouracil, cis-dichlorodiamineplatinum, and etoposide [15,16], and increased the DNA damage caused by CoCl_2_ or epirubicin [52]. These observations have been attributed to a reduced pentose phosphate pathway flux and, as a consequence, impaired DNA repair capacity. Additionally, high ROS levels promote DNA damage and genomic instability [53].

Despite applying four different shRNAs, we did not achieve a pronounced knockdown of *TIGAR*. Nevertheless, we obtained reproducible and consistent results. Moreover, as opposed to knockout models, the modulation of TIGAR we obtained was presumably more in the range of physiological changes, depending on, e.g., TP53. Overall, considering data from publicly available databases such as the Human Protein Atlas (www.proteinatlas.org) [54], and the R2 database (Genomics Analysis and Visualization Platform, http://r2.amc.nl), TIGAR does not seem to be a strong prognostic factor in glioma. The TIGAR-deficient mice developed quite normally, suggesting that compensatory mechanisms existed during development [37]. However, this does not exclude a relevant role of TIGAR in tumor biology, as transcription and translation are known to be differentially regulated depending on cell subpopulation and environmental conditions.

In the present study, we did not seek to identify the mediators of the effects observed upon *TIGAR* knockdown. Certainly, our results were based on a small modification of a complex network [55]. In accordance with previous reports [17], we found lower TKTL1 levels in LNT-229-shTIGAR cells than in control cells. However, this interference could not explain all the implications of *TIGAR* suppression outlined above. As a component of the acidic tumor environment, lactate can compromise immune surveillance, promote cell migration, and facilitate tumor progression [56,57]. Accordingly, *TIGAR* suppression, resulting in lower lactate levels, might contribute to a more oxidative and less aggressive phenotype. Both in normoxia and in hypoxia, extracellular citrate levels were elevated upon *TIGAR* knockdown. Citrate is synthesized as part of the tricarboxylic acid cycle and can be (1) further oxidized, (2) transported to the cytoplasm to serve as substrate for fatty acid and sterol synthesis or (3) released from astrocytes to the extracellular space [58]. Recently, a relevant impact of extracellular citrate on cancer cells has been demonstrated [59]. Exogenous pyruvate might help to maintain the proliferative capacities of hypoxic cells [60]. Reduced *TIGAR* expression could thus support tumor growth by raising levels of extracellular citrate and pyruvate. In addition, ROS in the tumor microenvironment not only affects tumor cells themselves, but also alters immune responses. Excessive ROS have been shown to compromise the activation, differentiation, and function of T cells, and to enhance T cell apoptosis [61]. Antagonizing TIGAR may also facilitate glioma progression by maintaining an immunosuppressive milieu. Hence, the mere inhibition of TIGAR function as part of a therapeutic strategy might have unexpected effects and cannot be considered advisable at present.

## 4. Materials and Methods

### 4.1. Reagents, Cell Lines and Culture Conditions

If not stated otherwise, reagents were purchased from Sigma-Aldrich (St. Louis, MO, USA) or Qiagen (Hilden, Germany). LNT-229 human malignant glioma cells, kindly provided by N. de Tribolet, LNT-229 cells stably expressing an shRNA targeting *HIF-1α* and its control (*Sima*), kindly provided by T. Acker [62], were cultured in Dulbecco’s modified Eagle’s Medium (4500 mg/L glucose), supplemented with 10% fetal calf serum (FCS; PAA, Pasching, Austria), 2 mM glutamine, 100 IU/mL penicillin, 100 μg/mL streptomycin and, if necessary (LNT-229-shHIF-1α and control cells), 10 µg/mL blasticidin at 37 °C and 5% CO_2_. In contrast to the LN-229 cells (ATCC CRL-2611), which display a *TP53* mutation at codon 98 [63], LNT-229 cells harbor a polymorphism in codon 72 and have transcriptionally active, wild-type TP53 [64,65]. The absence of mycoplasma contamination was verified before beginning experiments and bimonthly thereafter (MycoAlert^TM^ Mycoplasma Detection Kit, Lonza–Basel, Switzerland). For some experiments, glucose was added to the serum- and glucose-free medium to final concentrations of 2 or 5 mM. The shRNA sequences used were 5’-GCTGCTGGTATATTTCTGAAT-3’ TIGAR sh 97), 5’-GACAGCGGTATTCCAGGATTA-3’ (TIGAR sh 98) and scrambled control 5′-ACTACCGTTGTTATAGGT-3′ (scr sh), pGIPZ vectors encoded green fluorescent protein (GFP) and either scrambled shRNA (scr sh L1) or shRNA directed against *TIGAR* (TIGAR sh 2, 5’-AGGAAAAAATCACAGCTCT-3’, and TIGAR sh 3, 5’-TGGACAACCATATAGAATT-3’, Dharmacon, Lafayette, CO, USA). After transfection using Attractene (Qiagen), cells stably expressing shRNA constructs targeting *TIGAR* and scrambled control sequences, respectively, were selected by puromycin resistance (5 µg/mL). For transient overexpression of *HIF-1α*, pcDNA3 control (Invitrogen, Carlsbad, CA, USA) and pcDNA3-HIF-1α (Addgene, Cambridge, MA, USA) vectors were used. Hypoxia was achieved in Gas Pak pouches for anaerobic culture (Becton–Dickinson, Heidelberg, Germany). For irradiation experiments, cells, cultured in medium supplemented with 10% FCS and 25 mM glucose and highly proliferating, were exposed to single doses of 2 Gy photons using a linear accelerator (SL75/5, Elekta, Crawley, UK) with 6 MeV/100 cm focus-surface distance and a dose rate of 4 Gy/min. 0 Gy-controls were kept in parallel at ambient temperature in the accelerator control room.

### 4.2. SDS-PAGE and Immunoblotting

Cells were lysed in a buffer containing 50 mM Tris-HCl, 120 mM NaCl, 5 mM EDTA, 0.5% Nonidet P-40, 2 μg/mL aprotinin, 10 μg/mL leupeptin, 100 μg/mL phenylmethylsulfonyl fluoride, 50 mM NaF, 200 μM NaVO5, and phosphatase inhibitor cocktails I and II. Protein concentration was determined using a Bradford assay (Bio-Rad, Hercules, CA, USA). A total of 20 μg of total protein was separated under reducing conditions by sodium dodecyl sulfate polyacrylamide gel electrophoresis (SDS-PAGE) and electroblotted on nitrocellulose (Amersham, Braunschweig, Germany). Tris-buffered saline with 5% skim milk and 0.1% Tween-20 was used for blocking, primary antibodies (dilution 1:1000) applied were anti-TIGAR (polyclonal rabbit anti-human, ab37910, Abcam, Cambridge, UK) and anti-glyceraldehyde-3-phosphate dehydrogenase (GAPDH, monoclonal mouse, including but not limited to, anti-human, MAB374, Chemicon, Nuernberg, Germany). Immune complexes were detected by enhanced chemiluminescence (Pierce, Rockford, IL, USA). ImageJ was used for densitometric analysis according to http://rsb.info.nih.gov/ij/docs/menus/analyze.html#gels [66].

### 4.3. Real-Time Quantitative PCR (RT-qPCR)

TRIzol™ (Invitrogen, Carlsbad, CA, USA) and the RNeasy™ system (Qiagen) were used for the extraction of total RNA, 1.5 μg of RNA, and SuperScript VILO™ (Invitrogen) for cDNA synthesis. Real-time PCR was conducted in an iQ5 real-time PCR detection system (Bio-Rad, Munich, Germany) with ABsolute™ Blue qPCR SYBR-Green Fluorescein Mix (Thermo Fisher Scientific, Waltham, MA, USA). Gene expression data were normalized to the internal control succinate dehydrogenase complex, subunit A, flavoprotein variant (SDHA) using the ddCT method [67] and the iQ5 software (Bio-Rad). Primer sequences: HIF-1α forward 5’-GTCGGACAGCCTCACCAAACAGAGC-3’ and reverse 5’-GTTAACTTGATCCAAAGCTCTGAG-3’, SDHA forward 5′-TGGGAACAAGAGGGCATCTG-3′ and reverse 5′-CCACCACTGCATCAAATTCATG-3′, TIGAR forward 5′-CCTTACCAGCCACTCTGAGC-3′ and reverse 5′-CCATGTGCAATCCAGAGATG-3′.

### 4.4. ROS Analysis

In order to measure the ROS in living cells with intact membranes, we evaluated ROS levels at an earlier moment than cell death and provided slightly more glucose (5 mM). Cells were washed twice with phosphate buffered saline (PBS), incubated for 20 min at 37 °C with 10 µM dichlorodihydrofluorescein diacetate (H_2_DCFDA, Invitrogen), washed with PBS, and examined in a flow cytometer (BD Canto II, Heidelberg, Germany).

### 4.5. Growth and Viability Assays

Crystal violet was employed for the analysis of cell density. After the solubilization of the dye in 0.1 M sodium citrate, the absorbance was measured at 560 nm (Multiskan™ EX, Thermo Fisher Scientific, Langenselbold, Germany). For the quantification of cell death, adherent and non-adherent cells were collected, stained with 1 µg/mL propidium iodide (PI) and analyzed by flow cytometry. PI-positive cells were considered dead. Clonogenic survival was studied by seeding 500 cells in six-well plates, observing them for 7 days, staining them with crystal violet and counting colonies of more than 50 cells. Cell proliferation was quantified based on the incorporation of bromodeoxyuridine (BrdU Cell Proliferation ELISA, Roche Diagnostics). Assays evaluating acute growth inhibition or cell death required higher concentrations of temozolomide than those estimating colony formation, as previously described [68].

### 4.6. Luciferase Reporter Assay

Metafectene Pro (Biontex, Munich, Germany) was used to cotransfect LNT-229 cells with a 3HRE-pTK-luc [69] and, for the normalization of transfection efficiency, a pRL-CMV Renilla luciferase construct. The activity of Firefly and Renilla luciferase [70] was measured in an Infinite^R^ M200 PRO microplate reader (Tecan, Maennedorf, Switzerland).

### 4.7. Measurement of Extracellular Levels of Pyruvate, Lactate and Citrate

The cells were seeded in 6-well-plates, allowed to adhere overnight and cultured for 8 h in a serum-free medium containing 2 mM glucose. The diluted medium samples were then analyzed by gas chromatography coupled to mass spectrometry (GC-MS), as described previously [71].

### 4.8. Statistics

Experiments were repeated at least three times with similar results. Results were depicted as mean + standard deviation (SD), differences were considered significant if *p* < 0.05 using the two-tailed Student’s t-test.
